# Review of Novel High-Entropy Protective Materials: Wear, Irradiation, and Erosion Resistance Properties

**DOI:** 10.3390/e25010073

**Published:** 2022-12-30

**Authors:** Ana C. Feltrin, Qiuwei Xing, Akeem Damilola Akinwekomi, Owais Ahmed Waseem, Farid Akhtar

**Affiliations:** 1 Division of Materials Science, Luleå University of Technology, SE 97187 Luleå, Sweden; 2 Plasma Science and Fusion Center, Massachusetts Institute of Technology, Cambridge, MA 02139, USA

**Keywords:** high-entropy, wear, irradiation, erosion

## Abstract

By their unique compositions and microstructures, recently developed high-entropy materials (HEMs) exhibit outstanding properties and performance above the threshold of traditional materials. Wear- and erosion-resistant materials are of significant interest for different applications, such as industrial devices, aerospace materials, and military equipment, related to their capability to tolerate heavy loads during sliding, rolling, or impact events. The high-entropy effect and crystal lattice distortion are attributed to higher hardness and yield stress, promoting increased wear and erosion resistance in HEMs. In addition, HEMs have higher defect formation/migration energies that inhibit the formation of defect clusters, making them resistant to structural damage after radiation. Hence, they are sought after in the nuclear and aerospace industries. The concept of high-entropy, applied to protective materials, has enhanced the properties and performance of HEMs. Therefore, they are viable candidates for today’s demanding protective materials for wear, erosion, and irradiation applications.

## 1. Introduction

The crystal structure of a material determines its properties, and in-depth knowledge of its compositions and characteristics can lead to understanding its performance and efficiency in different applications. The traditional alloying concept is premised on adding relatively small quantities of secondary elements to a primary element to synthesize an alloy with enhanced properties. In the past two decades, a new paradigm shift in alloying concept has been developed [1,2]. This concept combines several primary elements in relatively high concentrations, often in equimolar concentrations, to synthesize new materials called high-entropy alloys. It has been proposed that high mixing configurational entropy favors the formation of simple crystalline phases over intermetallic compounds [1,2,3]. High-entropy materials (HEMs) have attracted ample attention in the materials engineering field due to their excellent properties, which are promising for diverse engineering applications. Besides, this new alloying concept has opened an opportunity to explore a vast realm of compositions and discover new alloys of scientific significance [3]. HEMs are commonly defined as materials having configurational entropy larger than 1.5 *R* (*R* is the ideal gas constant) [4], and are single-phase solid solutions, but this concept has been expanded to include multi-principal element alloys (MPEAs) and complex concentrated alloys (CCAs) and ceramics (CCCs), that can be multi-phase materials [3,5,6]. The relative enhancement in their properties is attributed to the chemical and structural disorder inherent in the high-entropy phase [7], which is premised on four core effects. These include high configurational entropy, sluggish diffusion, severe lattice distortion, and cocktail effects [8,9].

The first research on high-entropy alloys (HEAs) appeared in 2004 [1,2]. In the same year, researchers demonstrated the application of HEAs, in the form of nitrides, as advanced coatings. These studies have culminated in the development of various research fields, including high-entropy ceramics (HECs) [10], high-entropy composites [11], and high-entropy polymers [12]. With the rapid development and intense interest in this field, it is envisaged that future developments may encompass high-entropy dichalcogenides, high-entropy hydroxides, and high-entropy metal-organic frameworks [13].

HEMs can be built as bulk or coatings with many potential applications. They have exhibited superior temperature stability and thermal properties [14], good mechanical resistance and hardness [15,16], high oxidation resistance [17], excellent corrosion properties [18], radiation resistance [19], excellent wear resistance [20,21], electrochemical and electromagnetic properties [22,23], potential biomedical applications [24], and high erosion resistance [25]. Applying HEMs under extreme conditions is an important issue that must be addressed. 

**Figure 1:**
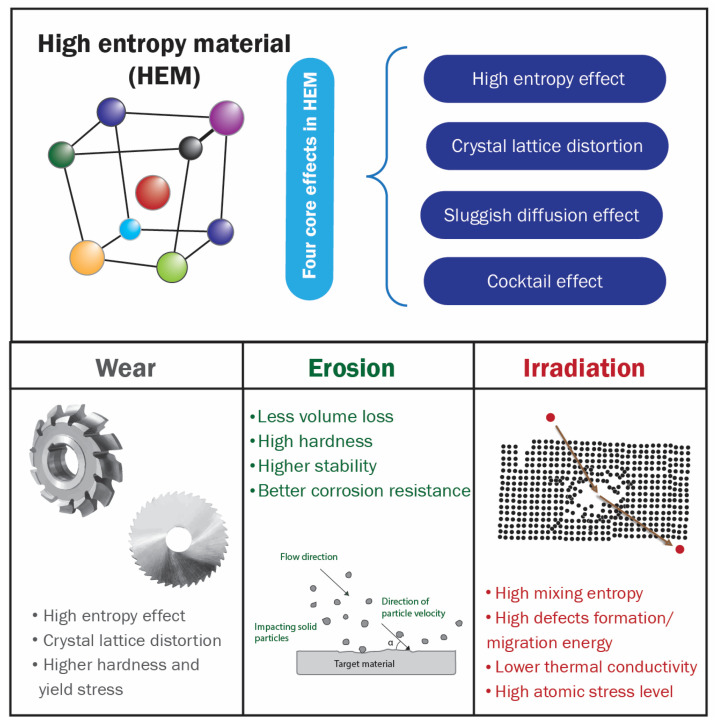
Properties of high-entropy materials that contribute to wear, erosion, and irradiation resistance applications.

This work reviews some critical aspects of applying high-entropy materials under extreme conditions vis-à-vis their tribological behavior, irradiation, and erosion resistance. This paper has been divided into four main sections. The first section briefly conceptualizes the high-entropy effect and its thermodynamics descriptors. Sections two and three address the mechanisms responsible for high-entropy materials’ enhanced irradiation and wear resistance. Section four describes the erosion resistance of HEMs. Each section concludes with a summary of key findings related to each application area, a future outlook, and possible research directions. Finally, the main characteristics of HEMs are listed and linked to these applications in Figure 1.

### 1.1. High-Entropy Concept

The development of new HEMs will open new application fields such as energy storage, gas storage, and sensing, superconductors, catalysts, and protection materials against irradiation, wear, erosion, etc., because improved properties may be observed. This happens because, unlike conventional alloys with one main element with small amounts of others, high-entropy alloys, for example, have more than four principal elements. Moreover, with slight variations in the chemical composition of HEMs, their microstructure, phase distribution, and properties can be altered [26], opening a wide range of applications.

The Gibbs free energy (Δ*G_mix_* = ∆*H_mix_* − *T*∆*S_mix_*, where ∆*H_mix_*, ∆*S_mix_*, and *T* are enthalpy and entropy of mixing, and temperature, respectively) is the reaction that predicts chemical reactions. As systems generally settle into low-energy states, new materials will be thermodynamically favored if the Gibbs free energy is negative [27]. On the other hand, the enthalpy effect is generally dominant, and intermediate products can be formed when mixing more than two elements [23]. Therefore, maximizing the entropy of the system is essential because when the entropy term overcomes the enthalpy term, the Gibbs free energy will be lowered, which may stabilize the solid-solution state relative to multiphase microstructures [28]. Besides, three other core effects (lattice distortion, sluggish diffusion, and cocktail effect) have been proposed to significantly influence the formation and properties of HEMs [27]. 

The representation of the synergy of HEMs is due to several parameters, such as the group of elements used in the composition, the particle size of starting materials, preparation methods, bulk or coating synthesis, etc. and the interaction between thermodynamics and kinetics, high-throughput computation and experimental work [15].

### 1.2. Development and Types of HEMs

#### 1.2.1. Alloys

**Figure 2:**
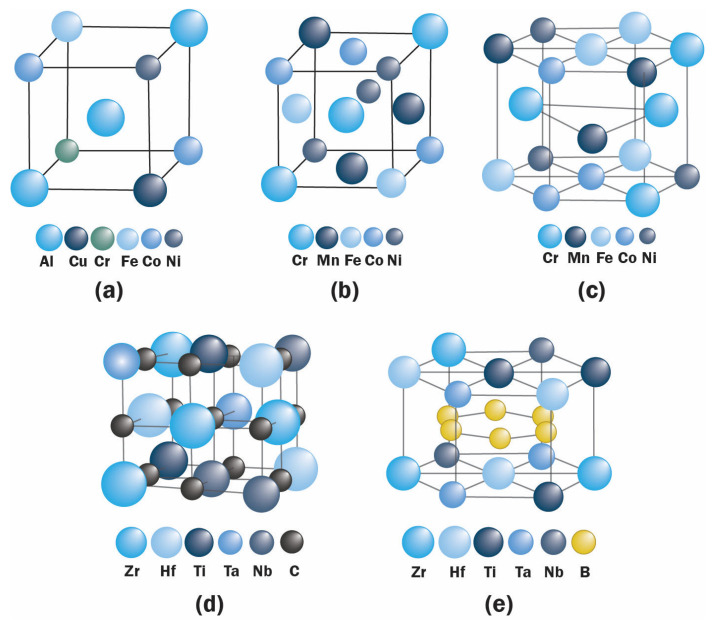
Proposed arrangement of atoms in the structures without considering lattice distortion of: (**a**) BCC CuCoNiCrAl_2.8_Fe [1], (**b**) FCC FeCrMnNiCo [2], (**c**) hcp CoCrFeMnNi [50], (**d**) rock-salt (Hf_0.2_Zr_0.2_Ti_0.2_Ta_0.2_Nb_0.2_)C [48], and (**e**) layered hexagonal (Hf_0.2_Zr_0.2_Ti_0.2_Ta_0.2_Nb_0.2_)B_2_ [27].

HEAs were introduced in 2004, culminating in an exponentially growing field of research. The goal of the high-entropy mixing system in alloys was generally directed to increase strength and ductility. The first developed composition is the CoCrFeMnNi alloy, termed Cantor alloy [2], formed in a face-centered cubic (fcc) system followed by other compositions with body-centered cubic (bcc) and hexagonal close-packed (hcp) structures, as exemplified in Figure 2a–c. 

The primary production method of bulk HEAs is by powder metallurgy, using mechanical alloying followed by spark plasma sintering (SPS), hot pressing, or hot isostatic pressing (HIP) methods. More detailed information is presented in the review by Torralba et al. [29]. Additive manufacturing (AM) is also used for bulk HEAs production, using laser metal deposition (LMD), selective laser melting (SLM), and particular electron beam melting (SEBM). The main advantage of bulk HEAs produced by AM is the complex geometrical shapes and structure tailoring. A review of HEAs produced by AM is presented by Moghaddam et al. [30]. A way to take advantage of HEAs is by applying them as films and coatings for conventional materials. It is better economically, and a good combination of costs and properties can be achieved [31]. In the same way, HEAs coatings and films have great potential in solving the demand for high-performance surfaces applied in extreme conditions [32].

The deposition of HEA coatings can be carried out by different methods, such as the sputtering deposition technique [33,34], resistance seam welding [35], laser cladding [36], electrodeposition [37], etc. Due to the high heating temperature and fast cooling rate, the microstructure of deposited HEAs is homogeneous, and the formation of amorphous and nanocrystalline phases is observed [32].

#### 1.2.2. Ceramics

HECs is a young research field, and its concept was created even before the definition of its name. After the description of HEAs in 2004, high-entropy nitride was synthesized in the same year [10], and in 2012 the term *high-entropy alloy oxide* appeared [38], describing the HECs family of materials. Nowadays, there are families of high-entropy nitrides [39], high-entropy carbides [40], high-entropy oxides [41], high-entropy borides [27,42], and more recently, high-entropy silicides [43], high-entropy borocarbides [11,44], and high-entropy oxyhalides [45]. Ceramic protective films and coatings are applied in industries as an alternative to bulk materials. The characteristics of high-entropy diborides, carbides, and nitrides as coatings and films have been reviewed and detailed elsewhere [42,46,47].

The crystal structure of HECs is characterized by the atomic disorder of metal elements occupying the cation position and non-metal elements occupying the anion position, resulting in compositional complexity and lattice distortion. The principal difference between HEAs and HECs is due to the lattice distortion that, in the first, occurs in the whole crystal structure, while in the latter, it occurs through the anion sublattice [48].

An Ideal HEC should have a lattice with long-range periodicity but compositional disorder. The reported formed structures of HECs are mostly single-phase rock-salt structure and layered hexagonal single-phase, as shown in Figure 2d,e. However, amorphous structure and dual-phase HECs have also been reported [49].

The SPS or other methods that apply pressure and temperature simultaneously are the primary preparation method for bulk high-entropy ceramics. On the other hand, the most adopted technique for coatings and films is the sputtering deposition technique.

Generally, synthesizing HECs aims to develop materials with improved electrical, thermoelectrical, and optical properties and as coatings on cutting tools and hard-facing materials. Among the different HECs, the (TaNbHfTiZr) carbide and nitride structures [48,51,52,53] have gained much attention as high-hardness coatings, high oxidation, and wear-resistant materials with good thermodynamic properties.

#### 1.2.3. Other Types of HEMs

*High-entropy composites:* The addition of SiC as the second phase in HECs has been investigated. Wang et al. [54] prepared a high-entropy composite using (Hf_0.2_Ta_0.2_Zr_0.2_Ti_0.2_Nb_0.2_)C doped with SiC to improve oxidation resistance at high temperatures. A high-entropy B_4_(HfMo_2_TaTi)C with SiC composite was synthesized, and it was proved that the addition of the secondary phase (SiC) offered oxidation resistance at high temperatures [11]. The addition of 20% SiC improved the mechanical properties of the high-entropy (Ti_0.2_Zr_0.2_Hf_0.2_Nb_0.2_Ta_0.2_)C, and the primary toughening mechanism was considered to be the crack deflection by the SiC [55].

*High-entropy polymers:* An HEA-polymer composite was synthesized and presented high specific strength and excellent recoverability under compression [56], showing the potential field to induce future studies with this brand-new material. Huang et al. [57] also used the concept of high-entropy to prepare a polymer blend with five different polymers, which manifested excellent performance, suppressing phase separation in the material.

*High-entropy metallic glasses:* The first report on high-entropy metallic glass was made by Ma et al. in 2002 [58], and its general properties are discussed by Wang [59]. The high-entropy metallic glasses were processed to combine the advantages of HEAs and metallic glasses, as higher thermal stability and sluggish crystallization process [60], enabling the use as high-performance materials. For example, they can have a high application potential under extreme irradiation conditions [61].

*High-entropy interlayers:* Medium-entropy alloys can be added as an interlayer between Al and steel, forming a high-entropy interlayer during fusion welding by thermal diffusion of the metallic elements. This can help to avoid brittleness in the welded joint [62]. For example, Ding et al. [63] produced a CoCrFeMnNi high-entropy interlayer as a diffusion bonding of copper and titanium, and strong adhesion between the layers was achieved at temperatures from 800 °C to 900 °C.

*High-entropy compositionally graded materials:* Addictive manufacturing can be used for synthesizing compositionally graded materials by varying the composition of each layer of deposited material [64]. Gwalani et al. [65], for example, produced Al_x_CoCrFeNi high-entropy alloy, with x = 0.3–0.7 and obtained single phase FCC grains with elongated shapes (x = 0.3) and FCC with typical lamellar morphology of a eutectic (x = 0.7), in the same produced specimen. 

### 1.3. Thermodynamic Descriptors

Predicting stable phases, in terms of the number of equilibrium phases and their mole fractions, as a function of composition is crucial to developing and designing HEMs [66]. However, unlike traditional alloys, the number of constituent phases in HEAs does not increase with the number of constituent elements. Therefore, the number of phases in HEAs is considerably lower than the maximum number estimated by the Gibbs phase rule [67]. Consequently, some theoretical models and empirical/semi-empirical parameters, based on the Hume–Rothery rules for binary alloy systems, have been proposed for predicting phase formation and stability in HEAs. Some of these parameters include enthalpy of mixing (Δ*H_mix_*) and entropy of mixing (Δ*S_mix_*) [3,68]. Others are atomic size difference (*δ*), valence electron concentration (*VEC*), atomic packing/gamma factor (*γ*), and *Ω* that relates the average melting temperature (*T_m_*) of a HEA with its Δ*H_mix_* and Δ*S_mix_* [69,70,71]. Although these parameters are primarily successful in predicting the formation of a solid solution in HEAs, some experimental discrepancies have been reported [72,73,74,75]. Nevertheless, they are essential starting points in the design and formulation of HEAs. A brief description of each of these parameters is presented in the following.

#### 1.3.1. Configurational Mixing Entropy, ΔS_mix_

In the early days of HEAs, the stabilization of a single-phase solid solution over the formation of an intermetallic or amorphous phase in HEAs has been primarily attributed to a dominant high configurational mixing entropy, Δ*S_mix_* [76]. The rationale is that the high mixing entropy contribution to the total free energy in alloys may stabilize the solid-solution state relative to multiphase micro-structures [28]. This criterion is derived from the number (*N*) of alloying elements with atomic concentration (*c_i_*), which increases the value of Δ*S_mi_*_x_ and lowers the Gibbs mixing free energy ($\Delta G_{mix}=\Delta H_{mix}-T\Delta S_{mix}$). The configurational mixing entropy, Δ*S_mix_* is expressed as in Equation (1).

(1) $$\Delta S_{mix}=-R\overset{N}{\underset{i=1}{\sum }}c_{i}\;ln\;c_{i}$$

Thus, it is postulated that a low value of Gibbs free energy suppresses the formation of ordered phases and intermetallics during solidification in the alloys [69]. Furthermore, calculations based on a hybrid genetic algorithm-molecular dynamics method suggest that Δ*S_mix_* plays a significant role in the comparative stability of the BCC or FCC phase [77]. 

However, this criterion has been criticized because it fails to predict the formation of multiple solid solution phases and other intermetallic phases in some HEAs [3,68,78]. For instance, Cantor [78] shows that Δ*S_mix_* increases significantly only up to N = 10. Beyond this value, there is only a minimal increase in Δ*S_mix_*, suggesting its limited contribution [68]. Similarly, ab initio calculations indicate that other entropic entities, such as vibrational, electronic, and magnetic entropy, significantly influence phase stability prediction [79]. Experimental evidence in [72] indicates that multiple solid solution phases are formed when the constituent elements in a model equiatomic CoCrFeMnNi HEA are substituted one at a time with another element having the same room temperature crystal structure and comparable size/electronegativity. The authors conclude that Δ*S_mix_* may not be the only dominating factor that governs phase stability [72]. Other experimental evidence reported in the work of Zhu et al., concluded that Ni and Al were responsible for phase stabilization for (CoCrFeMn)_(100-x)_Ni_x_ and (CoCrFeAl)_100-x_Ni_x_ alloys, respectively [73]. 

#### 1.3.2. Atomic Size Difference (δ) and Mixing Enthalpy (ΔH_mix_)

From the classical Hume-Rothery rules, two important factors significantly influence the formation of solid solutions in binary alloys. These are the atomic size difference (δ) and enthalpy of mixing (Δ*H_mix_*) [69]. It is postulated that a solid solution is unlikely to be formed when the *δ* of the constituent elements of an alloy exceeds 15% and Δ*H_mix_* is negative [66]. Consider an alloy system with *N* number of elements, *c_i_* the atomic percentage of the *i*th element, and *r_i_* is the atomic radius of the *i*th element, expressions for the atomic size difference (*δ*) and enthalpy of mixing (Δ*H_mix_*) are indicated below in Equations (2) and (3) [66,69]:

(2) δ=∑i=1Nci1−rir¯2

(3) $$\Delta H_{mix}=\overset{n}{\underset{i,j=1}{\sum }}4c_{i}c_{j}\Delta H_{ij}^{mix}$$

where $\bar{r}=\overset{n}{\underset{i=1}{\sum }}c_{i}r_{i}$ is the average atomic radius and $\Delta H_{AB}^{mix}$ is the mixing enthalpy of binary liquid alloys of the *i*th and *j*th elements. 

Accordingly, Zhang et al. [69] propose the following quantitative criteria for the formation of simple solid solution 12 J/(K mol) $<\Delta S_{mix}$ < 17.5 J/(K mol); −15 kJ/mol $<\Delta H_{mix}<$ 5 kJ/mol; and δ < 6.5%. Later, Guo and Liu modify the above criteria by including the data of equiatomic amorphous phase forming alloys to discount the effect of relative amount of each constituent elements [80]. The authors purpose the limits 0 ≤ δ ≤ 8.5%; −22 kJ/mol ≤ $\Delta H_{mix}$≤ 7 kJ/mol; and 11 ≤ $\Delta S_{mix}$ ≤ 19.5 J/(K mol).

It is clear from the above criteria that solid-solution phase formability is more relaxed than that proposed by Zhang et al. [69]. Nonetheless, it should be noted that these empirical parameters or criteria are not absolute in giving a correct prediction of the formability of a solid-solution phase. For example, there are overlapping areas where bulk amorphous phases, multiphase, and intermetallics are formed despite these criteria being satisfied [66,80]. Several experimental studies also report deviations from these criteria, where multiple phases and intermetallic compounds are formed [73,74,75].

#### 1.3.3. Valence Electron Concentration (VEC)

The valence electron concentration (VEC) is the total number of electrons and *d-*electrons in the valence band. It is a physical parameter that determines the stability of some solid solution phases. VEC is defined in Equation (4) [70]:

(4) $$VEC=\overset{N}{\underset{i=1}{\sum }}c_{i}{\left(VEC\right)}_{i}$$

where $c_{i}$ and ${\left(VEC\right)}_{i}$ are the the atomic percentage and valence electron concentration of the *i*th element.

Studies have shown that VEC, as a predictive parameter, has a weak influence in predicting phase stability, especially between amorphous or solid solution phases [74,75,80]. However, it is a useful parameter for delineating between BCC or FCC-type solid solutions. Generally, a high VEC favors the formation of the FCC phase, while a low VEC favors forming a BCC-type solid solution [74,75]. For this, Guo and co-workers suggest that for a VEC ≥ 8, only the FCC phase will exist. Conversely, a mixed FCC and BCC phases will appear when VEC is in the range 6.87 ≤ VEC < 8.0, and a sole BCC phase is expected when VEC < 6.87 [70]. Although some overlaps may be seen in distinguishing FCC/BCC phases, generally, these formula work for most HEA systems containing transition metals. The only exception is for alloys containing Mn [70]. Other experimental evidence implies that the VEC rule may not be suitable for the CoCrFeNi alloy [73]. For HEAs with the HCP phase structure, recent works by Takeuchi et al. on refractory IrRhRuWMo HEAs suggest that HCP phase formation can be reasonably predicted when 7.5 ≤ VEC ≤ 8.4 and VEC ~ 3 [81,82]. 

#### 1.3.4. Mixing Parameter (Ω)

Building on the earlier study of [66,69], Yang and Zhang [83] propose a mixing parameter (Ω) for predicting the formation of solid-solution in HEAs (Equation (5)).

(5) $$\Omega =\frac{\;T_{m}\Delta S_{mix}}{\left|\Delta H_{mix}\right|}\;$$

where $T_{m}$ is the average melting temperature of the alloy system, which is derived from the rule of the mixture as $\overset{N}{\underset{i=1}{\sum }}c_{i}{\left(T_{m}\right)}_{i}$.

The significance of Ω is appreciated by considering the Gibbs free energy expression. A positive value of Ω indicates that the contribution of *T*Δ*S_mix_* outweighs $\Delta H_{mix}$ at the melting temperature, hence a solid solution is expected to form. Conversely, when $\Delta H_{mix}$ dominates, Ω assumes a value less than unity, so intermetallic compounds are predicted [83]. By combining δ and Ω, the following limits are proposed for the formation of a solid solution phase [66,83]: Ω≥ 1.1 and δ≤ 6.6%. 

#### 1.3.5. Gamma (γ) 

The classical Hume–Rothery rules are premised on the distinguishable size difference between solute and solvent atoms in binary alloys. This size difference is, however, not strictly applicable to HEAs as constituent elements may often have similar sizes, and there is no strict distinction between solute and solvent atoms. Furthermore, the atomic-size difference (δ) parameter sometimes fails to delineate between solid solutions and intermetallics [66,80]. Therefore, Wang and co-workers propose a new parameter, gamma (γ), related to the random atomic packing between the smallest and largest atoms in HEAs [71]. If $r_{S}$ and $r_{L}$ are the radii of the largest and smallest atoms, γ can be defined as the ratio between the solid angles of the smallest $\omega_{s}$ and largest $\omega_{L}$ atoms in a HEA (Equation (6)) [71].

(6) γ=ωsωL=1−rS+r¯22−r¯2rS+r¯22/1−rL+r¯22−r¯2rL+r¯22

Calculations and analyses of data from 95 HEAs indicate that the gamma parameter can reasonably distinguish between the formation of solid solution (γ < 1.175) and ordered (γ > 1.175) phases (intermetallic and amorphous). It is assumed that the glassy/amorphous phases are metastable and can be transformed into intermetallic phases when subjected to equilibrium processing [71].

#### 1.3.6. Entropy-Forming Ability (EFA)

Although HECs are a relatively new subset of HEMs, some efforts have been devoted to describing an important empirical descriptor required to predict their formation and stability [15,84]. Sarker et al. [15] propose a descriptor called the *entropy-forming ability* (EFA), which relates the formation of a high-entropy single-phase crystal to its energy distribution from metastable configurations above the zero-temperature ground state. A narrow energy spectrum is proposed to favor high randomness/entropy due to a low energy cost requirement for metastability (i.e., high-EFA). In contrast, a wide spectrum indicates a high energy cost favorable for forming ordered phases (low-EFA) [15]. 

Consider an energy distribution spectrum, $H_{i}$ characterized by its standard deviation, *σ*. If *σ* describes configurational entropy, *S*, therefore, smaller values of *σ* correspond to higher *S*. Then, the EFA is defined as the inverse of the standard deviation of the energy spectrum above the ground state at zero temperature. For an HEC system with *N* species, the EFA (measured in [eV/atom]^−1^) is mathematically represented as in Equations (7) and (8) [15]:

(7) $$EFA\;\left(N\right)\equiv {\left\{\sigma {\left[spectrum\;\left(H_{i}\left(N\right)\right)\right]}_{T=0}\right\}}^{-1}\;$$

(8) $$\sigma \left\{H_{i}\left(N\right)\right\}=\sqrt{\frac{\sum_{i=1}^{n}g_{i}{\left(H_{i}-H_{mix}\right)}^{2}}{\left(\sum_{i=1}^{n}g_{i}\right)-1}}\;$$

where *n*, $g_{i}$ and $H_{mix}$ are the total number of sampled geometrical configurations, their degeneracies, and enthalpy of mixing, respectively. $H_{mix}$ can be calculated by taking the average of the enthalpies of the sampled configurations as Equation (9):

(9) $$H_{mix}=\frac{\sum_{i=1}^{n}g_{i}H_{i}}{\sum_{i=1}^{n}g_{i}}\;$$

Experimental investigations show that the EFA has a high accuracy in predicting the single-phase formation of HECs [15,84]. Further works by Harrington et al. [84] show that single-phase HEC is formed when the EFA > 45–50 eV/atom, and EFA < 45–50 eV/atom favors the formation of multiphase HECs.

## 2. Irradiation Resistance of HEMs

Materials used in the nuclear and aerospace industries suffer long-term radiation damage from high-energy particles. Recently, some studies have shown that HEAs may be suitable for these applications due to their low-volume swelling and high-phase stability when irradiated. However, irradiation damage is a complex process and is not well understood. Researchers have speculated that electronic and nuclear interactions may be involved. In addition, nuclear interactions are believed to be responsible for structural damage [85]. Bulk HEMs and their films/coatings have been studied in the literature. HEM coatings and films could be categorized into metallic-based that are synthesized from metallic elements [86,87] and ceramic-based that comprise different non-metals and anions, such as nitrogen, oxygen, boron, etc. [88,89]. Research in this area is still developing given the limited number of published articles on this topic. In this section, we discuss some of the underlying mechanisms responsible for the enhanced irradiation resistance of HEMs and review the effect of irradiation on their microstructure and properties.

### 2.1. Irradiation Resistance Mechanisms in HEMs

#### 2.1.1. Local Atomic Configuration

**Figure 3:**
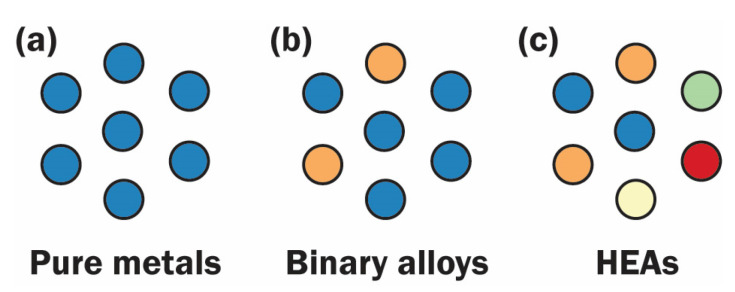
Comparison of the nearest neighbors around an atom among: (**a**) pure metals, (**b**) binary alloys, and (**c**) HEAs.

Unlike pure metals and binary alloys, atoms in HEAs are surrounded by neighboring atoms of different sizes, as illustrated in Figure 3. Thus, it is expected that the diffusion of vacancies or atoms will be significantly impeded, unlike in pure metals or binary alloys [90].

**Figure 4:**
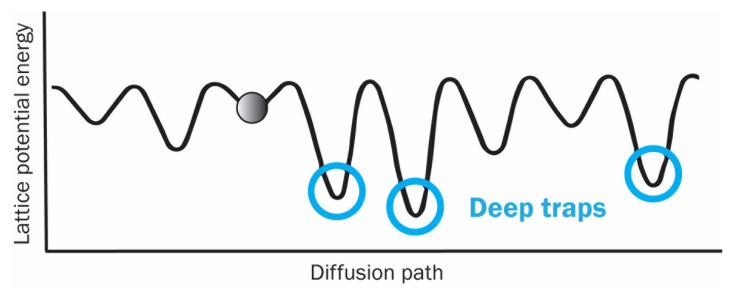
Schematic diagram showing the fluctuation of lattice potential energy along the diffusion path for an atom in the lattice (based on the figure of [90]).

Due to different surrounding atoms, the lattice potential energy of each atomic site is different. Therefore, in an irradiated HEA, an atom is not directly dislocated but experiences fluctuated lattice potential energy along its diffusion path in the lattice, as shown in Figure 4, circles mark deep traps. A larger fluctuation would cause a larger diffusion barrier. Such diffusion barriers are proposed to reduce the irradiation-induced defects in HEAs [90].

**Figure 5:**
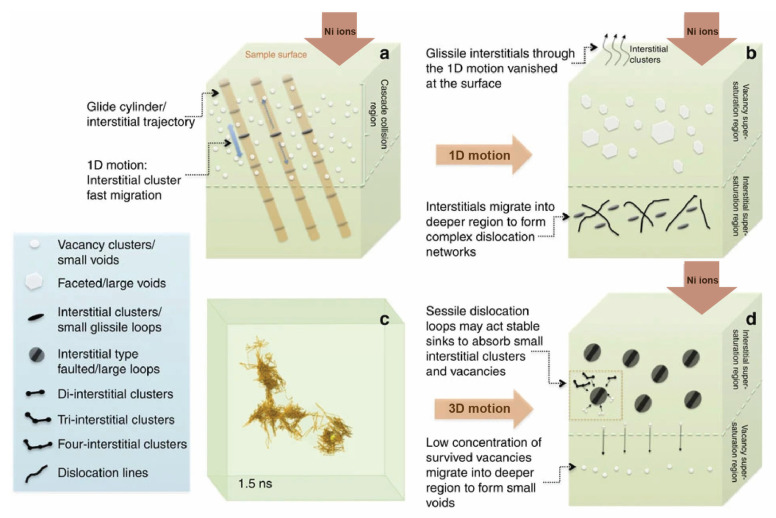
1D and 3D motions of interstitial clusters under ion irradiation. (**a**) Schematic illustration of 1D motion in pure metals. Interstitial clusters migrated fast along the glide cylinder. (**b**) Schematic sketch of defect evolution and distribution in pure metals (**c**) MD simulation result of the trajectory of the center of a four-interstitial cluster in HEA showing a 3D migration mode. (**d**) Schematic sketch of defect evolution and distribution in HEA [91].

Likewise, Lu et al. present some molecular dynamic calculations and experimental studies and propose that high-level site-to-site lattice distortions and compositional complexities in bulk HEAs can modify the diffusion pathways of irradiation-induced interstitial defects, such as vacancies, dislocation loops, and network dislocations [91]. They show that small clusters of self-interstitial atoms exhibit long-distance 1D migration without encountering and sweeping up a significant number of vacancies in the lattice of pure metals and conventional alloys. As these interstitial clusters migrate to longer distances, they leave a high-vacancy super saturation behind, which results in significant void swelling. Conversely, defect clusters migrate randomly in a 3D mode in HEAs. The suppressed 1D mode in HEA prevents interstitials from migrating rapidly out of the defect or damaged production region, which leads to reduced void swelling. Therefore, HEA shows enhanced resistance to irradiation damage or void swelling. The motion of defects clusters, in a long-range 1D mode in pure metals and short-range 3D mode in HEAs, is schematically shown in Figure 5 [91].

#### 2.1.2. Compositional Complexity

Some authors have proposed compositional complexity as one of the mechanisms responsible for enhanced irradiation resistance in HEAs. To reveal the relationship between radiation resistance and the configurational entropy, a conventional method is to gradually increase the component number of the alloys and then observe the evolution of radiation damage in them. The irradiation behavior of Cantor alloy and related nickel-containing single-phase alloy are extensively investigated by this method. For instance, studies on several nickel-containing single-phase equal atomic alloys support this proposition. By experiment and simulations, Granberg et al. [87] compare the radiation tolerance of pure Ni, NiFe, and NiCoCr bulk alloys. They notice that alloying slows down dislocation motion and reduces dislocation mobility, suppressing irradiation damage.

**Figure 6:**
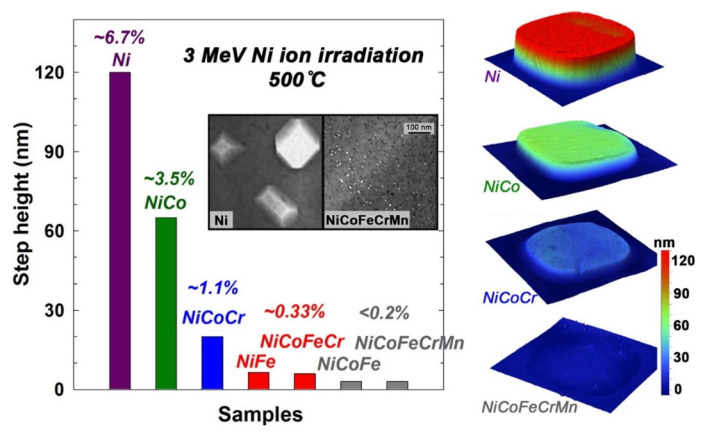
Influence of compositional complexity on radiation-induced volume swelling in Ni based alloys (reprinted from Ref. [93]).

Furthermore, Zhang et al. [92] investigate the effect of compositional complexity on bulk Ni, NiFe, NiCo, and NiCoFeCr alloys. Figure 6 shows the volume swelling of nickel-containing alloys after irradiation under 3 MeV Ni^+^ ion at 500 ℃. There is a significant decrease in the step height of the alloys as compositional complexity increases. Furthermore, volume swelling in bulk NiCoFeCrMn is smaller than in pure nickel [93].

In summary, the irradiation resistance has a positive relationship with the chemistry or compositional complexity of the materials.

#### 2.1.3. Atomic-Level Pressure

Another theory, based on atomic level pressure on atoms of bulk HEAs, has been presented by Egami et al. [94]. They demonstrate by first-principle calculation using the density functional theory approach that strong atomic-level pressures are present in HEAs not only due to the differences in the intrinsic atomic sizes, but also due to charge transfer among the elements reflecting the differences in electronegativity. For instance, in CoFeNi alloy, the atomic level pressures for Co, Fe, and Ni are −2.11, −15.07, and 18.75 GPa, respectively [94]. Similarly, the atomic level pressures of the elements in CoCrFeNi alloys are Co (9.01 GPa), Cr (−37.55 GPa), Fe (−5.74 GPa) and Ni (35.16 GPa) [94]. Positive and negative values represent compression and tension, respectively. 

The atomic level pressure in HEAs is higher than in conventional alloys. The local volume strain due to these pressures is of the order of ±0.1 for CoFeNi and ±0.2 for CoCrFeNi. The consequence of the atomic-level pressure is that they make amorphization of the alloy by particle irradiation easier. 

#### 2.1.4. Vacancy-Interstitial Defects and Localized Melting

**Figure 7:**
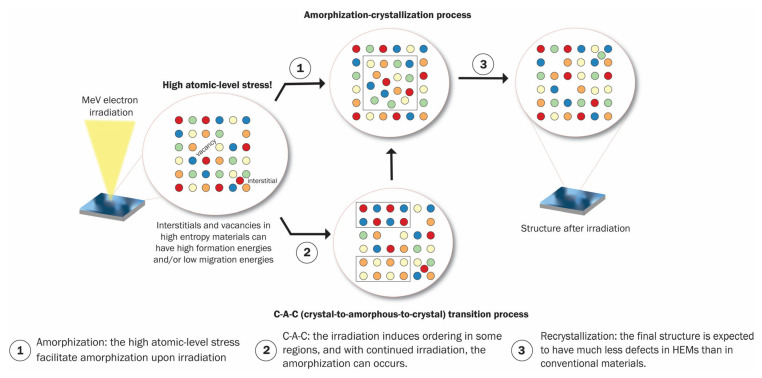
Schematic illustration of the recovery process in HEMs after going under irradiation (based on the description of Nagase et al. [85]).

When a HEA is irradiated with high energy particles, atoms of the different constituent elements in the HEA are displaced from their mean positions, which leads to the creation of various types of vacancy-interstitial defects or amorphization [95]. Simultaneously, these high energy irradiating particles deposit a high amount of heat, leading to a localized thermal spike. Consequently, local melting and rapid crystallization occur. These two effects lead to high-atomic stress as a consequence of the formation of several Frenkel pairs (interstitial-and-vacancy defects) in HEA that impedes dislocation movement, enhancing the irradiation resistance of HEAs [85]. An illustration of this recovery process and possible pathways, including crystal-to-amorphous-to-crystal (C-A-C) transition before the amorphization-recrystallization process, is shown in Figure 7. 

Similarly, Wang et al. [96], through the thermodynamic analysis, show that the equilibrium vacancy concentrations and their clusters in HEAs are substantially larger than those in pure metals and simple binary alloys. A large number of equilibrium vacancy concentrations may accommodate interstitial atoms generated by radiation damage. These analyses indicate that the equilibrium vacancy concentration in the HEAs will be much higher than that in pure elements, providing a strong interstitial and vacancy recombination tendency. 

Some experimental results on sputtered ZrHfNb HEA coating show BCC phase stability after 10 displacements per atom (dpa) irradiation at 298 K. Additionally, irradiation-induced structural change depends on the irradiation temperature and dosage [95,97]. Further experimental investigations on bulk CoCuCrFeNi show high phase stability of FCC structure under fast electron irradiation in a wide range of temperatures from 298 to 773 K up to 40 displacements per atom (dpa) [85]. Similarly, Al*_x_*CoCrFeNi bulk HEAs are reported to possess lower volume swelling than traditional nuclear materials after 3 MeV Au-ions irradiation [98]. 

### 2.2. Radiation Damage in HEMs

#### 2.2.1. Radiation-Induced Defects in HEMs

The radiation induces defect clusters that include dislocation loops in HEMs. The unique features of HEAs can reduce defect mobility during irradiation and suppress the migration and diffusion of defects. The FeNiMnCr bulk HEA had a smaller size and higher density of defect clusters than FeCrNi ternary alloy after being irradiated at 400–700 °C [99]. The phase structure also contributes to the irradiation tolerance of HEMs. The density functional theory (DFT) calculation on VTaCrW shows that the larger lattice distortion in BCC HEAs may lead to a better irradiation resistance when compared with FCC HEAs [100]. The Al_x_CoCrFeNi (x = 0.1, 0.75, and 1.5) bulk HEAs with the disordered BCC and FCC phase had less defect cluster than the ordered B2-phase after irradiated with 3 MeV Au^+^ ions [101]. 

**Figure 8:**
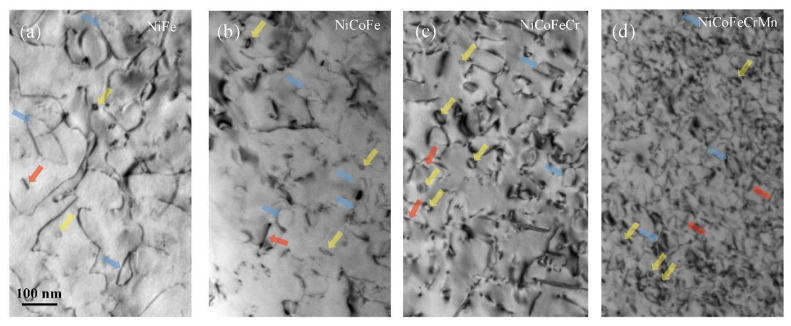
Dislocation loops in scanning transmission electron microscopy bright field images of nickel-contained equal atomic alloys irradiated to 38 ± 5 dpa at 773 K: (**a**) NiFe, (**b**) NiCoFe, (**c**) NiCoFeCr, and (**d**) NiCoFeCrMn. Perfect loops are marked by blue arrows, faulted loops are marked by yellow arrows, edge-on faulted loops are marked by red arrows (reprinted from Ref. [102]).

The stacking fault energy determines the type and morphology of the radiation-induced defects because low stacking fault energies suppress the formation of large size of dislocation loops in HEMs. Lu et al. [102] irradiated bulk NiFe, NiCoFe, NiCoFeCr, NiCoFeCrMn single-phase alloys with 3 MeV Ni^+^ ions at 773 K. The radiation-induced dislocation loops in the NiCoFeCrMn are smaller and denser than binary NiFe alloy (Figure 8) The growth of dislocation loops was delayed after increasing the complexity of compositions. Yang et al. [103] observed the delay of dislocation growth in the bulk NiCoFeCrPd HEA due to its higher lattice distortion. The low stacking fault energy suppresses the faulted dislocation loop into a perfect dislocation loop in FeCoNiCrTi_0.2_ bulk HEAs, which were rarely detected in the non-irradiated HEA [104]. The temperature is one factor that most affects the radiation-induced dislocation loops, and the mechanisms of radiation-induced segregation differ at different temperatures. Chen et al. suggested that the high-entropy and sluggish diffusion effects played little role in the dislocation-loop evolution at low irradiation temperatures for the Al_0.3_CoCrFeNi and CoCrFeMnNi bulk HEAs [105]. 

On the other hand, each element in the HEMs plays a different role in the radiation-induced damage. He et al. studied the element segregation in CrFeCoNi, CrFeCoNiMn, and CrFeCoNiPd bulk HEAs with 1250 kV electron irradiations at 400 °C to 1 dpa. The results indicated the Cr/Fe/Mn/Pd could deplete and the Co/Ni can accumulate at radiation-induced dislocation loops [106]. 

For bulk high-entropy ceramics, the first investigation was made by Wang et al. [107]. The microstructural evaluation was reported for (Zr_0.25_Ta_0.25_Nb_0.25_Ti_0.25_)C HEC under irradiation of 3 MeV Zr^2+^ ions (20 dpa) at 25, 300, and 500 °C. The lattice parameter increased 0.22% after irradiation at 25 °C and 0.19% after irradiation at 500 °C, and the irradiation-induced microstructures were identified mainly as dislocation loops. The growth of dislocation loops was being suppressed by the strong local lattice distortion caused by the high-entropy effect and the compositional complexity of the material that induces significant lattice distortion due to the presence of Zr, Ta, Nb and Ti atoms on the anion position.

#### 2.2.2. Radiation-Induced Cavities in HEMs

The formation of the cavities in the material’s microstructure is inevitable while the atoms are knocked away from their initial site. The cavities are concentrated with the diffusion of the atoms during irradiation and finally form macroscopic damages, such as helium bubbles and volume swelling. The suppression of radiation damage in HEMs is often attributed to the sluggish effect. The volume swelling of bulk Al*_x_*CoCrFeNi [98] and HfNbTaTiZr [108] are significantly lower than conventional alloys under similar irradiation conditions. The NiCoFeCrMn bulk HEA exhibited 40 times higher swelling tolerance than pure nickel, and the addition of iron has a stronger swelling suppression effect than chrome and cobalt in nickel contained alloys [93]. Yang et al. developed NiCoFeCrPd bulk HEAs with better swelling resistance than NiCoFeCrMn and they attributed the excellent swelling tolerance to the lattice distortion and higher defect migration barrier in this HEA [103]. 

Metallic materials irradiated by neutrons generate He^+^ ions because the transmutation and the concentration of vacancies generated can lead to He bubble formation. For instance, the FeCoNiCrTi_0.2_ bulk HEAs form He bubbles at the peak damage region after being irradiated by 275 keV He^+^ ions at 400 °C [104]. The low diffusion rate of the vacancy in HEAs is beneficial to suppress the He^+^ induced radiation damages. The number density of helium bubbles in Ti_2_ZrHfV_0.5_Mo_0.2_ bulk HEA is lower than traditional alloys irradiated on the same condition [109]. The average He bubble size in CrMnFeCoNi bulk HEA is smaller than pure nickel and 304 stainless steel after He-ion irradiation at 450 °C [51]. When irradiated with 6 keV He^+^ ions, He bubbles grow slower in FeCrMnNi HEAs than AISI 348 steel due to the sluggish diffusion effect in bulk HEA [110]. Chen et al. [111] found the He bubble volume fraction of FeCoNiCr bulk HEA is lower than pure nickel at the same irradiation condition. They proposed that the sluggish diffusion effect of He^+^ ion only works at high temperatures for the FeCoNiCr bulk HEA, and the He diffusion is affected by the featured point defects energetics [112]. Similarly, the suppression of He^+^ ion diffusion was also observed in HEA and HEC films. Pu et al. [113] irradiated nano-crystalline Al_1.5_CoCrFeNi HEA film at 300 K with 60 keV He^+^, and did not observe He bubble formation in the film, although He concentration was up to 8.50 at.% He bubbles were also not observed in AlCrMoNbZr/(AlCrMoNbZr)N multilayer coatings after being radiated with 400 keV He^+^ ions [114]. Structural and chemical stability of HEC coatings have also been demonstrated at high irradiation dosage. Komarov et al. reported structural and phase stability for (Ti, Hf, Zr, V, Nb)N coatings when irradiated with 500 keV of He ions [115]. As the irradiation resistance is directly related to thermal effects, which are connected to thermal conductivity and material’s thickness, the differences between coatings/films and bulk HEAs is expected to be different [116]. Although current studies on the irradiation resistance of HEA coatings and films appear to show similar behavior as their bulk counterparts, further studies are required to explicitly delineate this expected difference. More discussions about this have been covered in some previous studies [99,116].

### 2.3. Effect of Radiation on the Mechanical Properties of HEMs

Despite the thermal annealing caused by irradiation, the dislocation loops, voids, defect clusters, and precipitations induced by radiation will lead to the hardening and embrittlement of the materials. The mechanical properties of HEMs often have less variation than traditional materials due to their excellent tolerance to defect generation. On the other hand, self-healing and defect recombination in HEMs may decrease their hardness after irradiation. For instance, the nanoindentation hardness of Ti_2_ZrHfV_0.5_Mo_0.2_ [109] and HfNbTaTiZr [108] bulk HEAs had almost no increase after irradiation. The hardness of as-deposited W_38_Ta_36_Cr_15_V_11_ HEA film slightly increased after thermal annealing and after irradiation (1 MeV Kr^+2^ ions up to 1.6 dpa at 1073 K) [117]. A similar study on (TiHfZrNbVTa)N coatings reported enhanced mechanical properties after Au- ions implantation [89]. Another V_2.5_Cr_1.2_WMoCo_0.04_ bulk HEA demonstrated outstanding hardening and embrittlement resistance against 5 MeV Au^+^ ion irradiation of doses up to 42 dpa [118]. 

However, some HEAs show similar radiation hardening behavior to the traditional alloys despite their good resistance to radiation damage formation. The irradiation-induced hardening in FeNiMnCr bulk HEA is similar to conventional austenitic FeCrNi or FeCrMn alloys when irradiated with 3 or 5.8 MeV Ni^+^ ions at room temperatures [99]. Li et al. performed the neutron irradiation on NiFeMnCr bulk HEA at 60 °C up to 0.1, and 1 dpa, and the changes in the mechanical properties are similar to the austenitic stainless steels [119]. The hardness increase of Al_0.3_CoCrFeNi and CoCrMnFeNi bulk HEAs is slightly higher than 316H stainless steel after being irradiated with 1 MeV of krypton at 300 °C [120]. These results indicate that the mechanical behavior of HEMs under irradiation does not simply arise from the sluggish diffusion, and high configurational entropy, but from other mechanisms, such as the formation of dislocation loops in a short-range, which was also seen for stainless steel. 

For the bulk HEC studied by Wang et al. [107], nanohardness increased from 18.9 Gpa before irradiation to 20.5 Gpa after irradiation at 500 °C, because dislocation loops acted as barriers to the nanoindentations, impeding the slip and the lattice strain.

### 2.4. Future Scope

Although the chemical disorder is proven as an effective factor responsible for the enhanced radiation resistance of HEMs, it does not simply increase with the elemental number for traditional alloys. Besides the chemical complexity, the irradiation resistance of HEMs is also related to the species of alloying elements [93,121]. Therefore, the element selection needs to be discussed with the chemical disorder of HEMs in the alloy design. However, the elemental differences remain unknown and should be addressed in the future. In addition, high-activity elements such as cobalt and nickel are contained in the majority of HEMs. The replacement of these elements and its effect on the properties in the HEMs needs further study. The design of Co free HEAs attracted increasing attention in the development of radiation-resistant HEAs. Several low-activity HEAs, such as HfTaTiVZr, Ti_2_ZrHfV_0.5_Mo_0.2_, NiFeMnCr, etc., were reported to have superior irradiation resistance. Overall, more data on high-temperature irradiation is needed to understand the behavior of high-entropy materials, both bulk and coatings/films, in real irradiation conditions, to accelerate the development of radiation-resistant high-entropy nuclear structural materials.

## 3. Wear Resistance

The majority of HEAs are entropically-stabilized at high temperatures due to the mixing entropy effect [122]. Numerous publications focus on the tribology of HEMs, including abrasive wear, adhesive wear, and erosion wear behavior. The wear behavior of HEAs under different conditions, such as elevated temperature, wet environment, acid solution, etc., are also studied. Alloying, non-metalized, surface treatment, etc., were effective ways to increase the wear resistance of HEAs. In this part, we review the wear performance of three kinds of conventional HEMs, including HEAs, HECs, and high-entropy composites.

### 3.1. Wear Performance of HEAs

Structural materials are often required to have good wear resistance for reliable long-time service. Metallic glasses are promising materials in this area because of their high modulus and strength, and their amorphous coatings are widely applied on cutting tools. However, bulk metallic glasses are not suitable for high-temperature applications because crystallization may occur with the attendant loss of their desirable properties in the amorphous phase. The discovery of bulk HEAs and coatings offers an excellent solution to this problem.

#### 3.1.1. Wear Performance of Bulk HEAs

HEAs usually have excellent mechanical properties and thermal stability at high temperatures due to the high-entropy effect. These natural advantages make HEMs ideal candidates for wear-resistant and self-lubricating materials. The compositional adjustment is indispensable to increase the wear resistance of HEMs further. 

**Figure 9:**
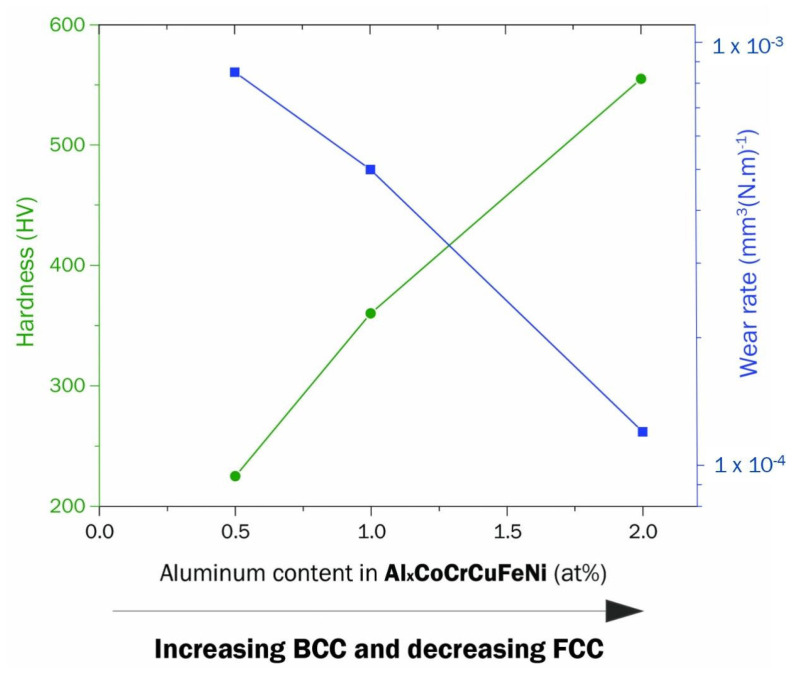
Effect on hardness and wear rate due to Al addition on Al_x_CoCrCuFeNi (based on the figure of Wu et al. [26]).

The composition adjustment may change the HEAs’ microstructure, and the formation of hard phase HEAs can improve the wear resistance. For example, the microstructure of Al*_x_*CoCrFeNi HEAs can transform from the FCC phase to the BCC phase with the increase in Al content [123], and the formation of the BCC phase can raise the hardness and reduce the wear rate [124]. Haghdadi et al. [125] studied the scratch behavior of as-cast Al_x_CoCrFeNi (x = 0.3, 1), and found the wear rate of Al_1.0_CoCrFeNi is lower than Al_0.3_CoCrFeNi. Zhao et al. [126] replaced the expensive Co with Al, and found the wear resistance of Al*_x_*CrCo_2-*x*_FeNi was improved with the increasing Al:Co molar ratio. This Al-hardening phenomenon was observed in other BCC + FCC duple phases HEAs, such as Al_x_CoCrFeNiMn and Al_x_CoCrCuFeNi. Cheng et al. [127] reported the wear rate of Al_x_CoCrFeNiMn dropped 63.7% at room temperature when x increased from 0 to 1. Besides the hardening, the study on the adhesive wear behavior of Al_x_CoCrCuFeNi [26] indicates that the oxidative wear caused by high Al content also plays a vital role in wear resistance improvement, as shown in Figure 9. Chuang et al. [128] studied the adhesive wear behavior of Al_x_Co_1.5_CrFeNi_1.5_Ti_y_ with different content of Al and Ti, and found that the wear resistance of the Al_0.2_Co_1.5_CrFeNi_1.5_Ti alloys is twice that of conventional wear-resistant steels SKH51. Besides Al, some researchers focused on the effect of other metals in CoCrFeNi based HEAs. Increasing Cu content can improve the wear resistance of the single FCC phase HEAs CoCrFeNiCu_x_ [129]. The addition of Nb increases the volume friction of laves phase in CoCrFeNb_x_Ni, and dramatically improves the wear resistance of the alloy [130]. 

The addition of nonmetallic elements may generate a hard ceramic phase in HEMs, which leads to an improvement in wear properties. For instance, surface boronizing can significantly increase the surface hardness of HEAs. The surface hardness of the Al_0.25_CoCrFeNi HEA increased from 188 HV to 1136 HV after boronized, and the wear rate is 12 times that in the unboronized HEA [131]. Lindner et al. reported the increased wear resistance of FCC-phase CoCrFeMnNi HEA after powder-pack boriding [132]. Nishimoto et al. [133] modified CoCrFeMnNi HEA using plasma nitriding, and the nitriding layer generated superior wear resistance of the HEA. The elements from group IV A can also enhance the wear resistance of HEAs. The wear resistance of FeCoCrNiW_0.3_ was as good as Co-based superalloys after adding 5 at.% carbon [134]. The wear resistance of CoCrCuFeNiSi_x_ HEAs can be improved with the increasing content of silicon [135]. 

#### 3.1.2. Wear Performance of HEA Coatings and Films

The addition of noble metals in traditional alloys is acceptable universally because the amount of the addition in weight percentage is small in most cases. However, materials will be uneconomical when these metals are designed as principal components. Therefore, coatings are an economical solution to provide surface properties such as corrosion or wear properties. The phase structure of HEA coatings tends to form an amorphous phase instead of the solid solution because of high cooling rates and may provide high hardness and good wear resistance. Jin et al. synthesized laser-cladded FeNiCoAlCu HEA coating on AISI 1045 steel substrate, and the friction coefficient decreased from 0.8–0.9 to ~0.3 when the temperature increased above 600 ℃ [136]. The mean friction coefficient of the surface of Q235 steel substrate reduced to 66% after laser-surface alloyed by FeCoCrAlCu HEA. The coating’s wear volume and specific wear rate were significantly lower than the substrate [137]. Guo et al. prepared MoFeCrTiWAlNb coatings using a rectangular-spot laser-cladding technique, which exhibited a smaller wear volume and lowers friction coefficient than the M2 steel substrate [138]. Shu et al. [139] studied the effect of amorphous content on the wear property of laser-cladded CoCrBFeNiSi HEA coating. In the coating, two layers were present, amorphous, and crystalline, while the higher amorphous content led to high hardness and better wear resistance. The two layers showed different wear mechanisms at high temperatures: the amorphous layer had mainly abrasive wear, while the crystallized layer had mainly adhesive wear. The amorphous layer had a lower wear rate when compared to the crystalline layer [140]. According to the research of Juan et al., the specific laser energy K plays an important role on the phase formation in laser-cladded HEA coatings as well [141]. 

As a promising metallic biomaterial, the Ti-6Al-4V alloy has poor wear properties. The wear resistance and coefficient of friction (COF) of Ti-6Al-4V alloy were enhanced after the deposition of TiTaHfNbZr HEA coating by magnetron sputtering [142]. Huang et al. [143] deposited laser-cladded dual-phase TiVCrAlSi HEA coating, which enhanced the wear resistance of Ti-6Al-4V alloy due to the combination of the complex (Ti, V)_5_Si_3_ phase and relatively ductile and tough BCC matrix. Wang et al. [144] prepared CuNiSiTiZr HEA coatings on TC11 alloy substrate via electro-spark and computer numerical control deposition. They found that the wear mechanisms of the CuNiSiTiZr HEA coating are cracking and delamination, which is different from the abrasive wear mechanism of the TC11 alloy. As a result, the substrate has a better wear performance after coating. Table 1 shows the details of experiments and different properties of some HEA coatings.

Several studies focused on the influences of single element content in HEAs on the wear behavior of the HEA coatings. Shu et al. [139] discovered the lower Fe to Co ratio increased the amorphous phase content in (Fe*_x_*Co_100-*x*_)_42_Cr_29_Ni_8_Si_7_B_14_ (*x* = 52, 57, 62, and 67). The higher amorphous content decreased the oxidation wear fraction, increased the coatings’ hardness, and enhanced the wear resistance at high temperatures. Wang et al. [145] added Ti into CoCrFeMnNi HEA coatings and investigated the tribology behavior at different temperatures. The tribological properties of the (CoCrFeMnNi)_85_Ti_15_ coating were 5.5 times those of CoCrFeMnNi coating at high temperatures. Wu et al. [146] prepared FeCoCrAlCuNi_x_ (x = 0.5, 1, 1.5) HEA coatings on the copper substrate using laser surface alloying, and the FeCoCrAlCuNi composition exhibited the lowest wear rate. Jin et al. [147] observed the phase transformation from FCC phase to BCC phase in FeCoCrNiAl_0.5_Si_x_ HEA coatings when increasing the silicon content. Due to the hardening effect of the BCC phase, the wear coefficient, wear depth and mass loss were reduced with the increasing content of silicon. 

Refractory elements can reinforce the wear properties of some HEA coatings. Cheng et al. [148] investigated the effect of Nb on the wear properties of CoNiCuFeCr HEA coating. After adding Nb, the wear resistance of the coating is about 1.5 times that of the coating without Nb under the same testing conditions. Wang et al. [149] compared the wear properties of MoFe_1.5_CrTiWAlNb_x_ (x = 1.5, 2, 2.5, 3) laser-cladded refractory HEA coatings. The results indicated that the higher content of Nb leads to lower wear volume loss in MoFe_1.5_CrTiWAlNb*_x_* HEA coatings.

### 3.2. Wear Performance of HECs

**Figure 10:**
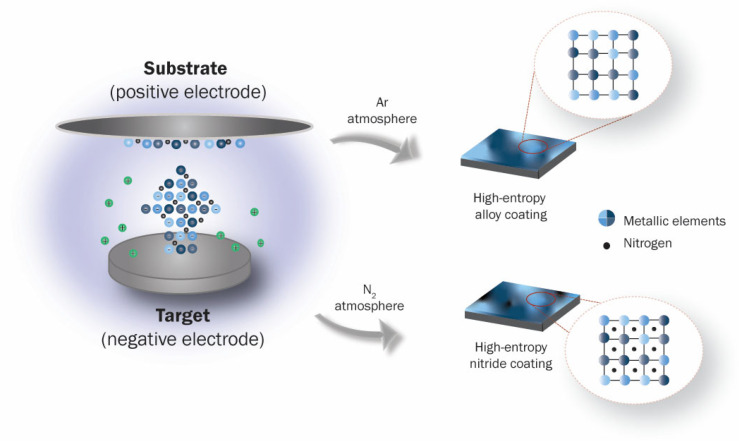
Different structures are obtained by magnetron sputtering using different atmospheres.

Magnetron sputtering is widely used in the manufacturing of HEA films. Mixing reactive gas as N_2_ into the deposition atmosphere is a convenient way to achieve HEC films during sputtering, as shown in the schematic Figure 10. Nevertheless, other deposition methods, such as vacuum arc deposition [89,150], have been reported. Sha et al. [149] investigated the effect of nitrogen on the microstructure and mechanical properties of (FeMnNiCoCr)N_x_ HEC coatings. They reported that its wear rate decreases for a higher content of nitrogen. Ren et al. [151] studied the tribology behavior of (AlCrMnMoNiZr)N_x_ HEC films deposited in different atmospheric conditions. The tribological properties of (AlCrMnMoNiZr)N_x_ worsen when the nitrogen flow rate increases. 

**Figure 11:**
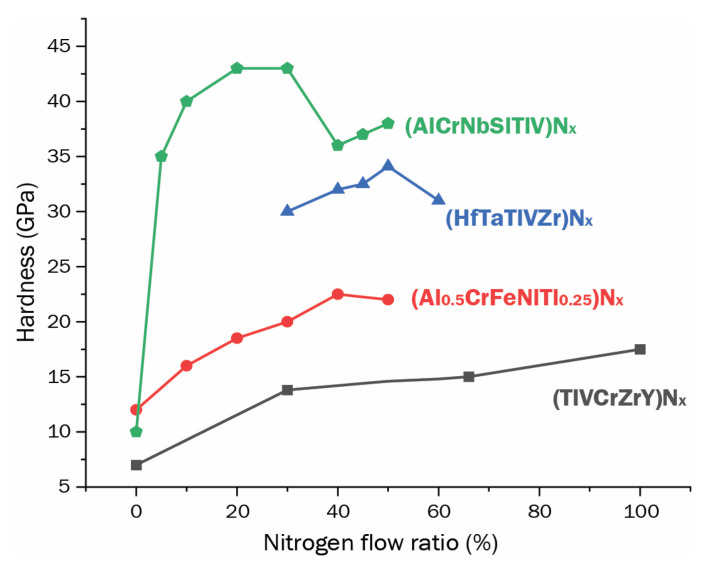
Influence of nitrogen flow ratio in the hardness of different high-entropy alloys coatings: (AlCrNbSiTiV)N*_x_* [152], (HfTaTiVZr)N*_x_* [153], (Al_0.5_CrFeNiTi_0.25_)N*_x_* [154], and (TiVCrZrY)N*_x_* [155].

Overall, the hardness of the films containing nitrogen increases with the increase in the nitrogen flow ratio until an optimum point before it starts to decrease. Some examples are shown in Figure 11. For (AlCrNbSiTiV)N_x_ developed by Huang and Yeh [152], hardness increased until a nitrogen flow ratio of 50%, where the maximum hardness was 41 GPa, placing the developed coating in the super hard class of materials. The increase in hardness was attributed to grain size strengthening, solution hardening, and residual compressive stress. For (HfTaTiVZr)N_x_ where nitrogen flow ratios were tested by Kirnbauer et al. between 30 and 60%, it was proved that the optimum flow ratio for increasing hardness was 45%, obtaining a hardness of 32.5 GPa [153]. Zhang et al. [154] developed (Al_0.5_CrFeNiTi_0.25_)N_x_, which presented higher hardness (21.78 GPa) at 40% of nitrogen flow ratio, while without nitrogen, the hardness of the coating was about 6 GPa. Although, for Tsai et al. [155], that developed the (TiVCrZrY)N_x_ coatings, the hardness increased even when the nitrogen flow ratio increased up to 100%, as well as the structure became denser due to grain growth and void elimination with the addition of nitrogen during the sputtering deposition, while the sample turned from NaCl rock-salt structure with 33% nitrogen flow ratio to amorphous at 100%.

**Figure 12:**
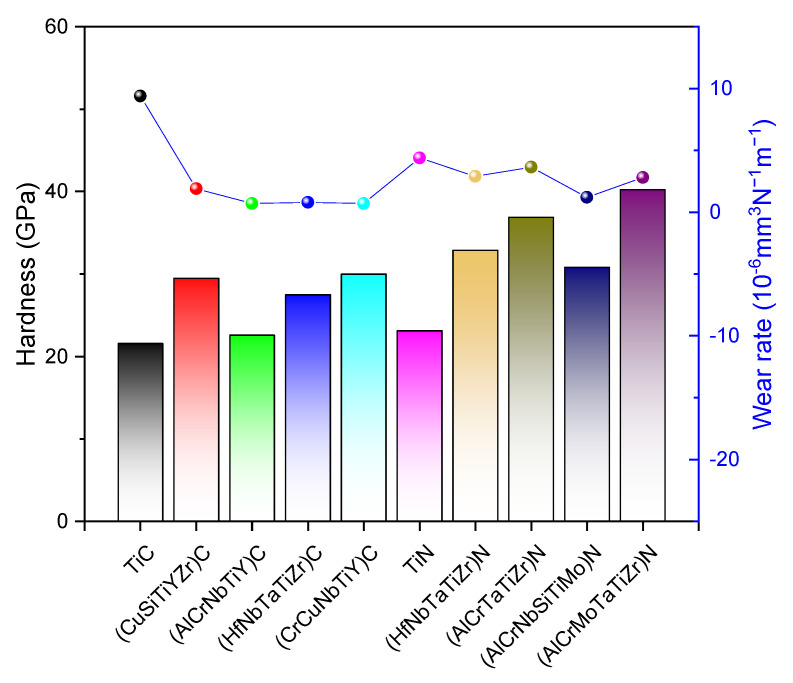
Hardness and wear rate of titanium-contained carbide and nitride films (prepared by magnetron sputtering) [24,52,151,156,157,158,159,160].

Braic et al. studied the wear behavior of a series of high-entropy carbide coatings deposited in a mixed atmosphere of argon and methane, such as (CuSiTiYZr)C, (TiZrNbHfTa)C, (TiAlCrNbY)C ,and (CrCuNbTiY)C [24,52,156,157,158]. Their research revealed that these HEC films exhibited superior wear behavior than traditional binary or ternary ceramic coatings. The COF of (TiAlCrNbY)C coating can be as low as 0.05. In addition, the wear resistance of the HEC films can be affected by the carbon content and substrate temperature. The amorphous (CuSiTiYZr)C coatings exhibit the lowest COF when the carbon/metal ratio is about 1.3. The (TiZrNbHfTa)C HEC coatings with high carbon content exhibit better wear behaviors than those with low carbon content. The (CrCuNbTiY)C coatings deposited at 500 °C and 650 °C presented better tribological behavior than others when substrate temperature was tested from 80 to 650 °C. Figure 12 compares the hardness and wear rate of some Ti-containing carbide and nitride films prepared by magnetron sputtering. It is notable that the high-entropy carbide ceramic films have higher hardness and lower wear rates than titanium carbide film. A similar trend was also observed in Ti-contained nitride films.

### 3.3. Wear Performance of High-Entropy Composites

The desire to develop high-entropy composites as wear-resistant materials is mainly based on two considerations. Firstly, hard ceramic particles, such as oxides, carbides, or nitrides are ideal reinforcements to increase HEAs’ hardness and wear resistance. Liu et al. [161] studied Y_2_O_3_ particles reinforcing CrMnFeCoNi HEA. The wear resistance of this HEA composite was improved due to the fine grain strengthening effect, and the composite exhibited the best anti-wear abilities for 0.25 wt% Y_2_O. Xu et al. [162] prepared graphene/CoCrFeMnNi HEA composites, and the graphene/HEA composite exhibited a lower COF and wear rate than the HEA matrix. The HEA powders can also be used as reinforcement in composite due to their high hardness and elastic modulus. Meng et al. [163] reinforced AZ91D metal matrix composites with AlCoCrCuFeNi HEA particles using the LMI method. They found both the wear volume loss and the specific wear rate were lower than unreinforced composites. Fang et al. [164] prepared Ti(C, N)-based cermets using Al_0.3_CoCrFeNi HEA as a metal binder. The Ti(C, N)-based cermets with CoCrFeNiCu metal binder were reported to have a remarkably lower wear rate than the Ti(C, N)-based cermets with Ni binders [165]. 

The second considerations are that the HEAs have the potential to serve in extreme conditions, such as high temperature or cryogenic environments. In these cases, the traditional lubricating systems are difficult to be applied, therefore, the self-lubricating property is necessary. Some solid lubricants, such as MoS_2_, graphite, etc., were introduced into HEAs to achieve self-lubricating properties in high-entropy composites. Liu et al. [166] reported that the COF of Fe_50_Mn_30_Co_10_Cr_10_ reduced by 61.9% after adding 0.2 wt.% graphene when under the load of 5 N. Zhang et al. [167] studied a series of self-lubricating CoCrFeNi HEA matrix composites prepared by SPS. The composite sintered from the CoCrFeNi HEA powder, Ni-coated graphite powder, and nickel-coated MoS_2_ powder had excellent self-lubrication and wear-resistance from room temperature to 400 °C, due to the synergetic lubricating effect and high-temperature oxidation. They also prepared composites using Ag, BaF_2_/CaF_2_ eutectic as the synergetic lubricants, which showed excellent self-lubrication ability from room temperature to 800 °C. 

High-entropy composite coatings can be obtained by cladding the mixture of reinforcement particles and HEA powders with micro and nano size. Peng et al. [168] fabricated FeCoCrNi HEA coatings with WC-reinforced particles. The composite coating with a high WC proportion of 60% had the minimum volume wear loss, which is better than Ni_60_/WC coating with the same WC content. The wear resistance of AlCoCrFeNi HEA composite coatings reinforced with NbC particles dramatically increased wear resistance due to the fine microstructure and the solution-strengthening effect [169]. Guo et al. [170] prepared in situ TiN particle-reinforced CoCr_2_FeNiTi_x_ (x = 0, 0.5, 1) HEA coatings. The wear performance of the coatings was improved after adding Ti atoms. Meng et al. [171] prepared AlCoCrCuFeNi/Mg composite coating using the laser melt injection method. The Cu rejection improved the wear properties of the coating due to increasing the ratio of H/E, but the formation of the brittle CuMg_2_ phase adversely influences the wear properties of the composite coatings.

### 3.4. Future Scope

The low wear rate in HEMs is attributed mainly to their improved mechanical properties, while the excellent high-temperature wear performance is attributed to their thermal stability and high temperature soften resistance. Because of the oxidation resistance, the worn surface of HEMs often forms deficient oxide layers. These oxide layers can reduce the COF of HEAs when sliding at high temperatures, although some issues still need to be addressed. For instance, temperature-related serration behavior was observed on the COF curves of HEMs, and the studies on the serration behavior can help to reveal the underlying wear mechanism of HEMs. In addition, introducing solid lubricants dramatically improves the high-temperature lubricating properties of HEMs, however, studies on novel self-lubricating high-entropy composites are still less explored. In view of the potential tribological performance combined with the structural complexity and broad range of compositions, HEMs can be used in several different applications in order to minimize wear.

## 4. Erosion Resistance

Erosive wear occurs when solid particles entrained in a fluid (slurry) strike the surface of a material, bringing many problems in industrial operations [172], promoting degradation and reducing the efficiency in flow components, i.e., turbine blades, pipelines, fluid machines, etc. Many parameters can affect this damage mechanism, such as the material’s structure, flow conditions, solid particles in the slurry, nature of the fluid flow, and temperature [173,174].

The erosion by slurries is also defined as an erosion-corrosion mechanism. It can be controlled by erosion (mechanical), which depends predominantly on the hardness of the material, or for corrosion (chemical or electrochemical), which depends on the chemical stability of the material. This concept is defined as synergy, which describes the wear rate experienced by a material under erosion-corrosion, and it is higher than only the sum of pure erosion and pure corrosion [174] as defined by Equation (10).

(10) $$V_{S}={\;V}_{T}-\left(V_{E}+{\;V}_{C}\right)$$

V_S_ is the volume loss of synergism, V_T_ is the total volume loss, and V_E_ and V_C_ are the volume loss by erosion and corrosion, respectively [175]. For corrosion on HEMs, the mechanisms were studied by several authors and are reviewed elsewhere [31]. The following section discusses the erosion mechanisms and experimental works performed with HEMs.

The erosion can deform, fracture, or completely displace the target material [176]. It is well known that there is a difference between the effect of erosion in ductile and brittle materials when the weight loss is measured as a function of the angle of impact, as the erodent particles can strike the material at different angles [172,177]. The erosion rate of a material is generally related to its hardness, which, when increases, is a beneficial factor in increasing wear, cavitation, and erosion-corrosion resistance [8,178]. However, other authors defend that for each structure can be a linear relationship between hardness and erosion wear resistance, but the microstructure of the material can have greater influence than the bulk hardness [179].

### 4.1. Erosion Resistance of HEMs

As the control of erosion wear is a complex process, there is a continuous interest in searching for new materials with superior mechanical properties [180]. However, the behavior of HEMs as erosion-protective materials has not been extensively explored [180] though research interest is increasing in this direction.

#### 4.1.1. Erosion Resistance of Bulk HEMs

The developed AlCrFeCoNiCu HEA [181] annealed was tested in a slurry pot erosion tester with a solution of 5 wt% of sand particles and 3.5% NaCl. The material showed higher chemical stability and better corrosion resistance after phase transformation of BCC before annealing to BCC + FCC after annealing. The mass loss after the slurry erosion test showed better performance of the annealed HEA than SS 304 in the same erosion conditions. The annealed HEA also showed a higher hardness when compared to the non-annealed HEA, proving that the mechanism of the erosion-corrosion, in this case, was due to the mechanical properties, not the chemical. The addition of Al in the Al_x_CrFe_1.5_MnNi HEA impaired the pitting resistance in chloride environments [182]. Tung et al. [183] affirmed that the formation of the BCC phase in AlCoCrCuFeNi HEA promoted an increase in the hardness due to the basic structure factor and solution hardening mechanism.

Nair et al. [180] compared the erosion-corrosion behavior of the Al_0.1_CrCoFeNi HEA (FCC) with SS316L. The test was performed using a re-circulation type test rig using a slurry of sand particles and 3.5 wt% NaCl solution. The HEA showed lower corrosion rates, and for the erosion, the HEA (H = 150 H_v_) presented a higher erosion rate than the SS316L (H = 226 H_v_) at 90° and 30° of angle impingement. For erosion-corrosion, at a normal impingement angle, both alloys showed nearly similar material removal. The mechanisms proposed were fracture and disintegration of the passive layer from the high-energy impact of the particles. While at 30° the HEA showed lower material loss compared to SS316L, which presented an increase in material degradation under the combined effect of erosion and corrosion. In addition, the authors described ploughing as the dominant material removal mechanism for HEA, while for SS316L was micro-cutting.

Kumar et al. [184] made an air-jet erosion test using alumina as erodent at four different angles on the Al_x_Fe_1.5_CrMnNi_0.5_ (x = 0.3 and 0.5) and described a higher erosion at lower impact angles due to plastic deformation, which happened due to sliding action of the erodent particles, occurring in the ductile mode of erosion. FeCoCrNi_2_Al HEA coating [185] was tested in High Temp Air Jet Erosion tester at 800 °C, and an increase in the depth of penetration of the erodent (alumina) increased with the temperature but did not pass through the coating, indicating that this HEA can be used as a protective material in high-temperature erosion applications.

#### 4.1.2. Erosion Resistance of High-Entropy Coatings and Films

The slurry erosion properties of Al_x_CoCrFeNiTi_0.5_ HEA coatings were first studied by Zhao et al. [186] using SiO_2_ particles as erodent material. With the increase of the Al in the composition, its hardness increased. The best composition tested was with x = 1, resulting in a good slurry erosion resistance in several impingement angles due to the high hardness, good plasticity, and low stacking fault energy of the HEA. Compared to the Cr_16_Ni_5_Mo alloy (widely used to fabricate hydraulic turbine components), all tested HEA compositions presented lower erosion rates at different impact angles. The addition of Al in the HEA promoted higher stability to localized corrosion because the Al formed a passive oxide film, protecting the HEA [187]. 

In a NiCoFeCrAl_3_ HEA coating [188], the largest volume loss was acquired at 45° after erosion with 15 wt% SiO_2_ particles in a jet erosion test machine, with a volume loss of 4.1 mm^3^. At the same time, in the same conditions, 17-7 PH SS (conventional wear-resistant steel) lost 5.4 mm^3^, showing that the HEA coating possessed better erosion resistance. The described erosion mechanisms were microploughing, microcutting, and removal of flakes. After annealing the coating, the cumulative volume loss was reduced because of the strength of the erosion resistance.

### 4.2. Improving Erosion Resistance of HEMs

The passive layer on the surface of the metal plays a dominant role in determining the erosion-corrosion behavior [180]. After testing SS in an acid medium, Aiming et al. [189] concluded that the passive film acts as a protective layer. When erosion removes the film, the material’s surface is exposed, and corrosion occurs. In the case of HEMs, Nair et al. [180] described the formation of a passive protective layer on the HEA material, which protected it from corrosion. Compared with a 316L substrate, the passive layer on the HEM was more stable, which enhanced its corrosion resistance. 

As mentioned before, the dominant mechanism in erosion-corrosion in two different works was ploughing and microploughing [180,188], suggesting a plastic deformation and abrasive wear on the surface of these materials while under erosion, which can be related to the hardness of the material, but further investigation of the mechanisms of surface degradation must be performed. Other authors proposed different mechanisms in common alloys [190], where the passive film is damaged by the particles, leading to a dissolution of the surface, which can remove the hardened layer and increase the roughness of the character, promoting bigger erosion rates, as the erodent particles can penetrate deeper into the surface. 

High-entropy coatings and films have drawn more attention for erosion/corrosion applications because they overcome the restrictions of preparation and high costs associated with HEMs. Besides, they can be applied to industrial parts that already exist, saving manufacturing costs and expanding their application ranges [191]. For future works, it is important to describe not only the influence of the surface hardness during erosion characterization. Rather, analyses should cover the role of the composition and microstructure of the HEMs together with their corrosion properties. In addition, research on high-entropy ceramics still ought to be addressed. Due to the high hardness and chemical stability of high-entropy carbides, borides, and nitrides, it is a potential field of new materials for erosion-facing materials. 

## 5. Summary and Outlook

The current state of the art summarized recent progress on the wear, irradiation, and erosion resistance of HEMs. A notable number of studies laid on the volume swelling, the mechanical properties, and the microstructural damages of HEMs after radiation. The effect of element addition, temperature, and lubricant on the wear resistance of HEMs draw extensive attention from researchers. 

The outstanding radiation and wear resistance of the HEMs are mainly due to high mixed entropy and lattice distortion. The hindrance to dislocation migration results in the high strength and high hardness of HEMs, which is beneficial to reduce the wear rate. The hindrance to atom migration increases the phase stability in high-energy environments, including high temperature and high-energy particle irradiation conditions. The inhibition of defect formation under the irradiation environment leads to low-volume swelling and low-irradiation hardening. In addition, the high-temperature oxidation resistance promotes the formation of deficient oxide on the worn surface, which may offer a lubricating effect to HEMs at high temperatures. 

The erosion resistance of HEMs was related mainly to their mechanical properties. However, in environments with erosion/corrosion, chemical properties also had influence, standing out the importance of the four core effects: high-entropy effect, lattice distortion, sluggish diffusion, and cocktail effect. Currently, more efforts were made towards developing and testing HEAs for wear, irradiation, and erosion properties. However, there is limited information on thermodynamic descriptors and characterization of high-entropy ceramics and composites, for example, towards these applications. Nevertheless, their potential properties indicate that more importance should be given to this class of HEMs.

## Figures and Tables

**Table 1:** Characteristics and wear properties of different high-entropy alloy coatings.

Preparation Method	Coating Composition	Coating Microstructure	Substrate	Temperature	COF	Ref.
Laser cladding	FeNiCoAlCu	BCC	AISI 1045	RT	0.8–0.9	[136]
				600 °C	0.3	
Laser cladding	FeCoCrAlCu	BCC	Q235	RT	0.87	[137]
Laser cladding	MoFeCrTiWAlNb	BCC + (Nb,Ti)C	M2 steel	RT	0.5–0.6	[138]
Laser cladding	TiVCrAlSi	BCC + (Ti,V)_5_Si_3_	Ti-6Al-4V	RT	~0.3	[143]
Magnetron sputtering	TiTaHfNbZr	Amorphous	Ti-6Al-4V	RT	0.1–0.2	[142]

## Data Availability

Not applicable.
